# Gebiss: an ImageJ plugin for the specification of ground truth and the performance evaluation of 3D segmentation algorithms

**DOI:** 10.1186/1471-2105-12-232

**Published:** 2011-06-13

**Authors:** Janos Kriston-Vizi, Ng Wee Thong, Cheok Leong Poh, Kwo Chia Yee, Joan Sim Poh Ling, Rachel Kraut, Martin Wasser

**Affiliations:** 1 Bioinformatics Institute, Agency for Science, Technology and Research (A*STAR), 30 Biopolis Street 07-01 Matrix, 138671, Singapore; 2 School of Biological Sciences, Nanyang Technological University, Singapore; 3 Department of Biological Sciences, National University of Singapore, Singapore; 4 Translational Research Resource Centre, Laboratory for Molecular Cell Biology, Medical Research Council, University College London, London, UK

## Abstract

**Background:**

Image segmentation is a crucial step in quantitative microscopy that helps to define regions of tissues, cells or subcellular compartments. Depending on the degree of user interactions, segmentation methods can be divided into manual, automated or semi-automated approaches. 3D image stacks usually require automated methods due to their large number of optical sections. However, certain applications benefit from manual or semi-automated approaches. Scenarios include the quantification of 3D images with poor signal-to-noise ratios or the generation of so-called ground truth segmentations that are used to evaluate the accuracy of automated segmentation methods.

**Results:**

We have developed Gebiss; an ImageJ plugin for the interactive segmentation, visualisation and quantification of 3D microscopic image stacks. We integrated a variety of existing plugins for threshold-based segmentation and volume visualisation.

**Conclusions:**

We demonstrate the application of Gebiss to the segmentation of nuclei in live *Drosophila *embryos and the quantification of neurodegeneration in *Drosophila *larval brains. Gebiss was developed as a cross-platform ImageJ plugin and is freely available on the web at http://imaging.bii.a-star.edu.sg/projects/gebiss/.

## Background

The widespread use of automated florescent confocal microscopy has resulted in a significant role for image analysis in modern quantitative biology. Quantitative features such as the number of cells or fluorescent intensity of subcellular organelles have become crucial for the elucidation of many biological and pharmaceutical hypotheses ranging from cell biology to anticancer drug development in various organisms such as *Caenorhabditis elegans *[1], *Drosophila melanogaster *[2-4] and even rodent models [5]. With the advent of three-dimensional (3D) optical sectioning of confocal microscopes and green fluorescent protein (GFP) as an expression marker [6], spatial distribution of cellular organelles can be studied. Histone tagged with fluorescent protein (e.g. GFP) [7] allow the observation of DNA distribution in living cells. Recent innovations in light sheet microscopy enabled the study of the spatiotemporal organisation of nuclei in whole zebrafish and *Drosophila *embryos [8-10]. A vast amount of visual information is acquired in automated microscopy. Some of the extracted features are less sensitive to the precision of segmentation, such as the number of objects and their location based on centroid coordinates. Other, biologically important features such as shape or volume require a more precise segmentation.

Mechanisms of cell cycle regulation can be elucidated by live cell imaging and subsequent automated quantification of nuclei in intact organisms [11]. The living *Drosophila *embryo provides an attractive experimental system for the study of mitosis, where nuclei can be observed *in situ *[12].

Neurodegeneration is another biological phenomenon of intense interest that has been subjected to extensive study in *Drosophila *models, but for which there are few quantitative cell biological readouts. Generalized brain neurodegeneration has been studied in *blue cheese (bchs) Drosophila *mutants [13], where ubiquitinated protein accumulation and a failure of degradative trafficking pathways have been implicated [14,15]. Kumarasamy et al. [16] determined by quantitative automated multivariate analysis of wide field fluorescence images that the degenerative phenotype was accompanied by changes in the size and distribution of lysosomal compartments within neuronal termini.

Loss of motor neurons has been documented in the third instar larval nervous system of *bchs *mutants, as well as superfical observations of smaller ventral ganglion size by confocal microscopy [13].

Image segmentation is an important step in the image processing workflow that is extensively applied in fluorescence microscopy. During segmentation foreground pixels are separated from background pixels. The use of machine segmentation (MS) in automatic image cytometry enables the measurement of cellular features in a high throughput fashion. However an automated imaging workflow cannot fully supplant the expertise of a trained biologist to detect and evaluate phenotypes. In a previous report, poorly-segmented cells were identified by eye in the framework of a high-content screening imaging pipeline [17]. Each of the authors of the report independently reviewed an equal fraction of the test image set, classifying into well-segmented and poorly-segmented qualitative groups using subjective criteria. Segmentation performance evaluation is still not common in cell-based high-content screening. Subjective descriptive terms such as "reasonably conformed perimeter" can serve well to train classifiers evaluating segmentations qualitatively and find features resistant (intensity-based features) or prone (morphology-based features) to imprecise cellular segmentation [17]. Besides "good" and "poor" segmentation, a quantitative evaluation can answer questions such as "how good" or "how poor" a machine segmentation algorithm is. The first step towards achieving such a quantitative evaluation is building a segmentation dataset that contains only well-segmented objects.

Performance evaluation methods can be divided into analytical and empirical groups, where the former investigates the algorithm directly and the latter judges it through the quality of the image segmentation. The empirical discrepancy method uses an ideal or expected segmentation result to objectively quantify the performance of an algorithm [18]. This concept takes into account the difference between an automatically segmented and a reference image and is generally used for practical image processing performance evaluation in real applications, where the accuracy of segmentation is the primary concern [19-23]. This approach produces easily interpretable results and is useful to perform a quantitative comparison of different segmentation algorithms. However, such a reference, or ground truth (GT) dataset creation is generally considered as a labour-intensive step [24], where human intuition or judgment makes an expected objective evaluation to be influenced by subjective factors. The GT is defined as a reference data set that acts as the gold standard in segmentation evaluation. In the context of image segmentation, the GT can be represented in various formats that are created manually or semi-automatically by human experts. Contours represent regions of interest, while labelled 2D and 3D images are the most comprehensive format that include all pixels and voxels of detected image objects. In specialised applications such as nuclear segmentation, the GT is encoded in the form of centroids [10,25]. A designer of an image analysis system has an array of often task-specific machine segmentation choices, where the GT is the "correctly" segmented image, which is needed for objective numerical evaluation of those algorithms. Since there is no unique universal segmentation ground truth, against which machine segmentation results can be compared, a human expert must create a perceptually consistent GT. Currently, there is no dedicated software tool to evaluate segmentation quality. A number of programs have been developed to segment and visualise 3D optical image stacks automatically or interactively. The potential software packages ranges from the commercially available Imaris (Bitplane AG, Zurich Switzerland), Definiens Developer XD (Definiens AG, Munich Germany) and Matlab (Mathworks, Natick USA) to open source alternatives as Cellprofiler [26], the Segmentation Editor plugin under ImageJ [27]/Fiji [28] and ITK-SNAP [29]. However, the use of these applications are limited in 3D segmentation performance metrics [30].

Here, we present a software for manual 3D segmentation and segmentation performance evaluation: Gebiss (Ground Truth Editing and Benchmarking for Image Stack Segmentation). Gebiss was developed as an ImageJ [27] plugin. We used Gebiss to assess the neurodegenerative state of *Drosophila *mutants by measuring brain volumes. Such a phenotypic readout would permit us to test the effects of pharmacologic or genetic interventions that may affect the severity of the phenotype. Gebiss [31] was applied to the analysis of over 5000 single images from different 3D image datasets.

## Implementation

**Figure 1:**
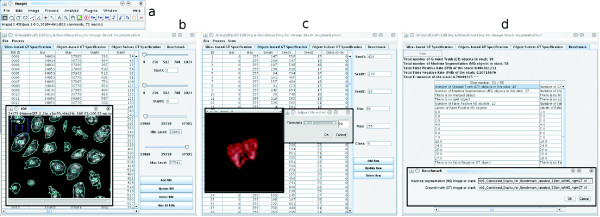
**Gebiss graphical user interface**. The standard ImageJ interface (a) is used to open an input stack before Gebiss is launched. The major modules of Gebiss divide the interface into tabs. The "Slice-based GT Specification" tab (b) is devoted to 2D ground truth creation, where each ROI of an object can be specified by a different threshold. 3D ground truth creation is possible using the "Object-based GT Specification" tab (c), which is coupled with a 3D visualisation window (inset). The numerical performance evaluation is implemented into the "Benchmark" tab (d), that prompts a MS and a GT labelled stack as an input (inset), and returns various performance measures in a tabular format.

Gebiss was developed as an ImageJ plugin to help the biologist to generate a ground truth. The source code availability, platform independence and wide developer base made NIH ImageJ [27] (Figure 1a) an optimal environment for a ground truth creation and benchmarking application. Gebiss leverages ImageJ's core capabilities such as easy installation, opening and saving in a wide range of image formats, such as Image Cytometry Standard ICS [32], uncompressed and ZIP-compressed TIFF stack or Zeiss LSM by the plugin OME LOCI Bio-Formats [33]. The graphical user interface of Gebiss (Figure 1b-d) was developed using the Swing toolkit for Java and its simple design guides the user along the workflow. Formerly an unsettled issue, memory allocation is not limited anymore. Both the current versions of Java and ImageJ are able to handle 64-bit platforms and > 4 GB RAM.

Besides ImageJ, Gebiss requires a Java3D installation for spatial visualisation. This can be done easily by following the steps of Benjamin Schmid's guide. An alternative to the standard ImageJ is Fiji [28] which contains Java3D as part of a package. The Gebiss installation itself consists of downloading gebiss_.jar and biiImageJ3DViewer.jar from http://imaging.bii.a-star.edu.sg/projects/gebiss/ and copying those into the "plugins" folder of ImageJ or Fiji, after which a "Gebiss" submenu appears automatically in the Plugins menu. Gebiss uses Jarek Sacha *ij-plugins Toolkit *that can be freely downloaded from http://ij-plugins.sourceforge.net/ or version 1.4.1 can be found at the project web folder that must be copied into the "plugins" folder of ImageJ or Fiji.

Gebiss is run after an 8, or 16-bit greyscale microscopic image stack is opened. The spatial voxel dimensions can be imported either automatically from an ICS, TIFF, or LSM stack header, or otherwise can be set manually in ImageJ. Gebiss provides a function to set and save voxel depth enabling the storage and repeated retrieval of a value over several imaging sessions.

The methodology is illustrated with examples of live, wild type *Drosophila melanogaster *embryonic nuclei monitored with GFP histone H2A variant (H2Av-GFP) in various phases of the cell cycle. The confocal microscopic image stacks of the anterior part of the embryo were captured by a Zeiss LSM 5 DUO microscope.

### Gebiss workflow

**Figure 2:**
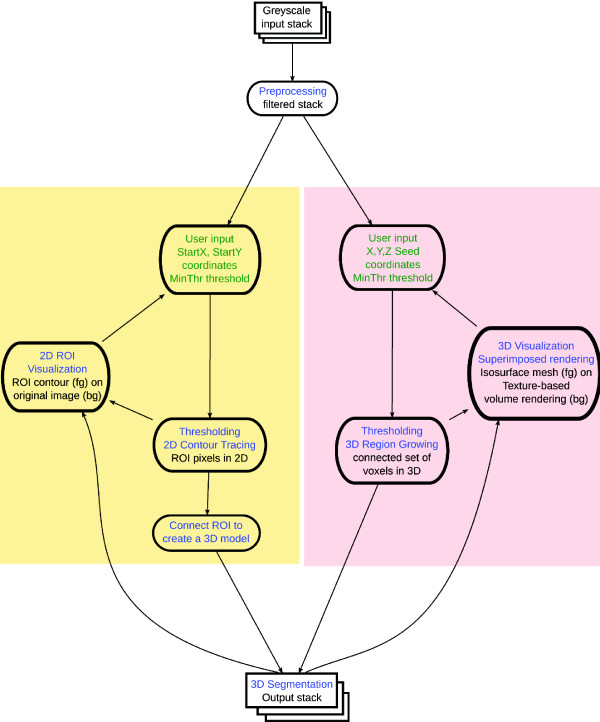
**Gebiss pipeline**. The diagram describes a typical 2D (left side) and 3D (right side) ground truth creation workflow with Gebiss. Green text indicates user interaction, blue text shows automated steps. Input and output data are written in black text. The steps are detailed in the text.

The generation of a labelled ground truth image stack in Gebiss can be achieved through: i) segmenting each 2D ROI of a spatial object, ii) 3D segmentation of a spatial object or segmenting a group of spatial objects (Figure 2). The labelled GT stack can be saved and used to benchmark an arbitrary machine segmentation. Each original microscopic image stack is smoothened by applying a 3 pixel radius (7 × 7 pixel window sized) two dimensional median filter.

#### 2D slice-based GT creation workflow

In the course of a slice-based segmentation (left side section of the flowchart in Figure 2), each optical slice of a spatial object can be segmented individually using different threshold values if necessary. The resulting ROIs can effectively exclude otherwise merged segmentation artefacts. Inspired by Michael M. Miller's SegmentingAssistant plugin and using ImageJ's core Wand tool class [34], each ROI is defined by four parameters: StartX, StartY, a minimum (*θ_min_*) and a maximum intensity (*θ_max_*) threshold value.

While SegmentingAssistant makes 2D local segmentation much easier and precise compared with a manual "freehand selection", there was room for improvement. For example, sliders are not perfect for StartX, StartY selection in order to assign several points in an image. A slider is not optimal to set *θ_min_*, when the typical step is too small to be set by dragging the slider. Also, the parameter load and save functions were missing. Earlier the binary mask and labelled stack creation was complicated, as it required the combined use of ROI Manager and the 3D object counter [35] plugin.

Several features were built in Gebiss to improve productivity. StartX and StartY coordinates can be assigned by a single mouse click on the original image and *θ_min _*can be changed by rolling the mouse middle scroll wheel in intensity steps of 20 and 50 when combined with Shift or Ctrl key respectively. The parameter *θ_max _*is set to the brightest intensity value of the image by default, and requires adjustment in rare cases. The algorithm searches in the right direction until a pixel is found with an intensity falling into the range of [*θ_min_*, *θ_max_*]. Following that, it traces the contour of the 2D object in a clockwise direction. However, the resulting ROI is calculated by using the median filtered intensity values, which are invisible to the user. Superimposed on the original image, users are guided with continuous visual feedback as the contours are updated. Using the GUI of the Slice-based GT Specification tab (Figure 1b), the optimal StartX, StartY, *θ_min _*and *θ_max _*values are stored in a parameter database for each object, where they can be updated, deleted or invoked to create a binary and a labelled stack.

The 2D slice-based GT creation workflow is flexible enough to allow the use of multiple threshold values per 3D object if needed. At the same time, the reuse of the stored parameters of an object in the present optical slice enables faster processing of the consecutive slice. The latter approach assumes that the StartX, StartY and *θ_min _*parameters of the object ROI in the previous slice gives a correct contour. If not, the values can be adjusted to fit on the given contour.

#### 3D object-based GT creation workflow

Gebiss is able to visualise 3D-thresholded foreground voxels as an isosurface superimposed on background voxels with original intensity. This double rendering feature can be switched on by checking in the Add isosurface submenu in the View menu of Gebiss, which appears when the tab is activated.

After an image stack is opened with ImageJ and Gebiss is started, 3D connected objects can be segmented interactively (Figure 2) by using the tab "Object-based GT Specification" (Figure 1c). Each 3D object is segmented individually by a seeded 3D region growing algorithm [36] originally implemented as ij-plugins Toolkit version 1.2 by Jarek Sacha. The Connected threshold region growing segmentation algorithm requires five parameters specified by the user: the x, y, z coordinates of a seed pixel as well as a minimum (*θ_min_*) and a maximum intensity threshold value. Following the right side section of the flowchart in Figure 2, the seed pixel coordinates are defined on the image stack window by a single Ctrl plus left mouse key click on a bright fluorescent region (where intensity greater than *θ_min_*) of a given object. The *θ_min _*is defined immediately after the seed selection.

To provide the user with 3D visual information to find the optimal *θ_min_*, a customized version of Benjamin Schmid's ImageJ 3D Viewer plugin [37] was implemented into Gebiss. In order to simultanously visualise background and foreground, Gebiss superimposes two 3D renderings. Background voxels are visualised by texture-based volume rendering using brightness-corrected fluorescent intensities. Foreground voxels are added forming a semi-transparent, red coloured isosurface mesh that allows the user to observe simultanously both the interior and the exterior of the object. The default maximum intensity threshold value is 255 for images with a dark background and a bright foreground. The user selects an optimal *θ_min _*value in the "Adjust threshold..." window (Figure 1c). In the case of *Drosophila *embryos such a *θ_min _*value is low enough to include all heterochromatin regions and chromosome arms but high enough to exclude free histone and lipid droplets. By dragging the threshold slider, a 10-15 slice thick 3D mesh of a typical nucleus is rendered. The image can be freely rotated in 3D and zoomed using the mouse and the middle wheel. The whole rotating 3D virtual environment can be recorded as an animated movie using ImageJ's AVI writer plugin.

An automated light attenuation compensation in GT creation is offered by a dual thresholding function implemented under the tab "Object Subset GT Specification". There are cases where the signal-to-noise ratio would allow the segmentation of a stack with a global threshold, though the light attenuation requires the use of a higher threshold value for deeply located objects. In this module two different thresholds are applied to objects located in shallower and deeper axial depth. The user is prompted for a data file containing the x, y, z seed coordinates of each object, a threshold value for the shallower and a second threshold value for the deeper objects as well as the demarcation z slice number that separates the shallow and deep slices. The x, y, z seed data file is generated using the 3D object counter [35] plugin. Even if its global thresholding segmentation produces imprecise contours in such cases, the derived object centroids are saved in a data file that serves as an x, y, z seed input file. This Gebiss module applies a 3D region growing segmentation using shallower or deeper *θ_min _*to the objects according to their z centroid value. In practice it is done as follows. A separation slice is set up at a certain depth. All nuclei are segmented by a 3D region growing algorithm. Those nuclei whose centroid's z parameter (the depth of the centroid point) is above the separation slice are segmented by a user-determined threshold value. Those nuclei whose centroid's z parameter is below the separation slice are segmented by a different threshold value.

#### GT contour visualisation

The requirement of human supervision for GT creation in any system may require an optional high level check. Fast and precise visualisation is achieved by the superimposition of all GT ROI contours on the original images. An ImageJ macro was created which uses a binary GT and original microscopic greyscale stacks as input files (see additional file 1: "ImageJ macro for GT contour visualisation"). The binary stack must be inverted (i.e. black foreground objects on a white background), and the original greyscale stack must be converted to RGB format. The macro automatically draws all segmented object contours on each of the original slices, visualising both the foreground and background pixels (see additional file 2: "3D GT contour visualisation"), therefore any missing 2D ROI or 3D object can be detected at the object level. At pixel level, false negatives such as unsegmented chromosome arms and false positives such as attached free histones or lipid droplets can be recognised easily. Any further GT ROI adjustment can be done by the respective Gebiss steps. The isosurface 3D rendering of a labelled stack can reveal falsely merged GT objects.

#### Benchmarking workflow

For segmentation performance evaluation, the most useful measures are precision, recall (sensitivity) and F-measure. All of those measures need the quantification of true positive (tp), false positive (fp) and false negative (fn) class labels defined by the four outcomes of the relation between the predicted class and the actual class. The value of *precision *is calculated as , thus it depends on the number of false positives. Fewer false positives result in a precision value closer to 1. Similarly, the number of false negatives affect the *recall *value, that is calculated as , which is closer to 1 when the number of false negatives is low. The *F-measure *is expressed as , and it evaluates the performance of a machine segmentation in a single value as the harmonic mean of precision and recall. The closer the F-value is to 1, the better the given MS is. A ground truth segmentation contains neither any false positive nor any false negative pixel, therefore *p_GT _*= 1, *r_GT _*= 1 and *F_GT _*= 1. In the context of segmentation, the performance evaluation can be quantified at two levels: object level and pixel level.

At the object level, the object number of MS and its deviation from that of the GT is calculated. The "object" term refers to either a 2D foreground region of interest (ROI) composed of 4 or 8-connected pixels or a 3D body composed of 18 or 26-connected foreground voxels. Ideally, each GT object matches an MS object resulting in a one-to-one correspondence. The machine segmentation may result in a false positive object that does not occur in GT or a false negative, missing one that does occur in GT. An MS object may be split (one-to-many correspondence) if more than one MS object matches a GT object or merged (many-to-one correspondence) if more than one GT object matches an MS object.

At pixel level, the correspondence between the foreground and the background region is quantified. Each segmented pixel or volumetric pixel (voxel) can be classified as true positive, false positive, true negative (tn) or false negative. The number of tp, fp, tn and fn pixels are counted, and precision, recall and F-measure are calculated for a 3D stack or a 2D slice. The benchmarking module requires a labelled MS and a labelled GT stack as input files. It has a numerical output (Figure 1d) and a visual output that is shown in additional files 3 and 4 ("Numerical output of the Gebiss benchmarking module" and "Visual output of the Gebiss benchmarking module", respectively). The numerical output displays benchmark measures for the whole stack as well as each individual slice. For the whole stack, the number of GT and MS objects are indicated at object level. Several well-established performance metrics [30] are in use (precision, recall and F-measure), that are derived from the confusion matrix. The precision, recall and F-measure are calculated at the pixel level. For each individual slice, the module calculates the number of GT and MS objects as well as the number and label of merged and split objects (if any). This feature enables the precise identification of the slice where ROI merge occurs. The number and label of occurrent false positive MS and false negative GT objects are also calculated.

## Results and Discussion

Gebiss is a tool for semi-automated 3D image segmentation which can be used either as a ground truth in performance evaluation or in image quantification. We applied it to different biological datasets. A good example is the process of nuclear division in the early *Drosophila *embryo, which has been extensively described. Precise nuclear segmentation is critical in quantifying this aspect of embryonic development. Contour finding in nuclear segmentation can be challenging as lipid droplets surround and attach to nuclei. The condensed chromosomes are often surrounded by a cloud of free histone that impedes the segmentation of a specific surface. This problem also applies to all deeply located nuclei in a stack where laser attenuation leads to poor signal-to-noise ratio. Additionally, the contrast of a whole image stack may be low owing to the uniform intensity of foreground and background pixels. Gebiss offers the biologist diverse ways to overcome these obstacles.

### Case study 1: Nucleus segmentation in embryogenesis

#### 2D segmentation of embryonic nuclei

**Figure 3:**
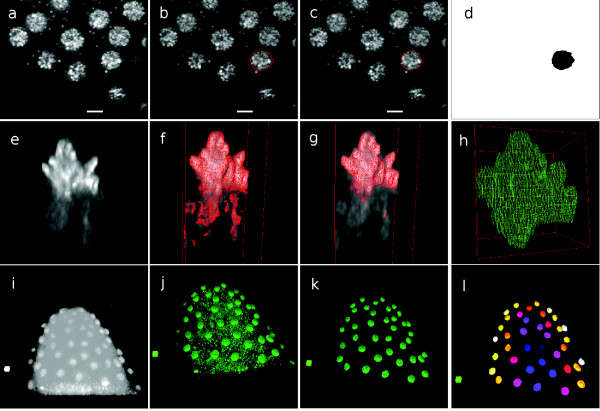
**Comparison between original and segmented data**. Original data (a, e, i). Segmentation with incorrect (b, f, j) and correct (c, g, k) threshold values. Segmentation results (d, h, l). **Top row: **To separate a lipid droplet from a nucleus, a 2D slice of the original image (a) is segmented through a contour resulted by an incorrect (b) and a correct *θ_min _*value (c), finally showing the segmented image (d). The scale bars indicate 5 *μm*. **Middle row: **3D segmentation of a metaphase nucleus (e) with (f) and without (g) free histone attachments and a 3D mesh isosurface representation of the segmented slices (h). The dimensions of the bounding cube are 7.1 *μm *× 8.8 *μm *× 13.6 *μm *(e-g), 10.5 *μm *× 12.6 *μm *× 7.8 *μm *(h). **Bottom row: **Low contrast image stack (i) segmentation leads to adding visually significant false positive voxels by using a global threshold (8-bit threshold value *θ *= 44) (j). It is segmented correctly by 3D object-based segmentation (k), resulting in a labelled stack (l). The regular cubes in the left side of the panels (i)-(l) indicate 50 *μm *^3^.

One of the objectives of this study is measuring the volume of nuclei. To measure the precise nuclear volume, segmentation must correctly separate an attached lipid droplet from a discrete one that locates in the close proximity of a nucleus. Gebiss can be first used to measure the volume of nuclei marked by H2Av-GFP. To quantify nuclear features like size, shape, number, etc., it is necessary to separate the two classes of nuclear histone and cytoplasmic histone. A lipid droplet-specific marker, such as Nile Red dye [38], effectively allows lipid droplet segmentation and separation from the nuclei, but using the less organelle-specific H2Av-GFP it is still possible to create a precise ground truth segmentation. The top row of Figure 3 illustrates the 2D slice segmentation of a nucleus optical section from the original image (Figure 3a) through a contour resulting from an incorrect (Figure 3b) and a correct (Figure 3c) *θ_min _*value. Figure 3d shows the final segmented image of the nucleus. Decreasing the *θ_min _*value for a whole 3D object is usually not helpful to detach a lipid droplet artefact because 3D shrinking leads to the loss of the correct spherical shape of an interphase nucleus. Consequently, we disconnected a lipid droplet by the *θ_min _*reduction of only certain 2D nuclear slices while we preserved the correct nuclear shape.

**Figure 4:**
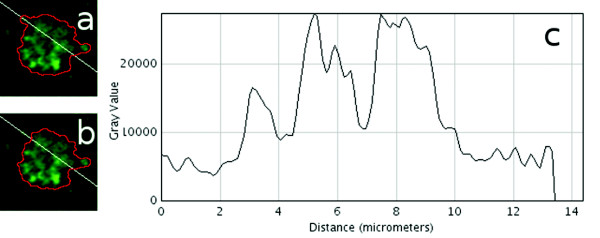
**Lipid droplet and nucleus segmentation**. Low H2Av-GFP intensity gradient in an optical section between a distinct lipid droplet and a nucleus. (a)-(b) 2D segmentation contours using 16-bit *θ_min _*= 9131 (a) and *θ_min _*= 9574 (b). The lipid droplet locates in the upper left region of the image, intersected by the white profile line. Size of the images (a) and (b): 10.8 *μm *× 10.8 *μm*. (c) Greyscale intensity profile plot. The intensity values of the lipid droplet and the nucleus range between 2 - 4 *μm *and 5 - 10 *μm *respectively.

The H2Av-GFP intensity gradient is often low between a distinct lipid droplet and a nucleus before the histone assembles into the chromatin (Figure 4). This complicates the 3D gradient-based segmentation. The intensity variation inside a nucleus can exceed that of the variation between the nucleus and a distinct lipid droplet nearby (Figure 4c).

The repeated use of a certain parameter set in multiple ROI slices allows accelerated GT creation, however it points out the intrinsic limitations of the method when used for segmentation of a spatial object. Certain ROIs can overlap each other and can be omitted inadvertently as a result of parameter reuse. Since only one slice is shown at a time and the thresholding parameter can be adjusted arbitrarily, the resulting contour can be flickery over slices of low contrast stacks. Finding the optimal thresholds of the top and bottom slices and the contours can be difficult in such cases. 3D segmentation alleviates those shortcomings.

**Figure 5:**
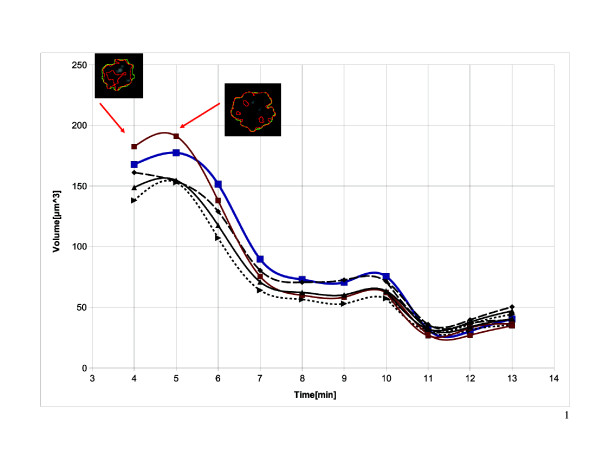
**Time-lapse mitotic volume change of a nucleus at syncytial blastoderm stage**. The volume of a representative syncytial blastoderm nucleus was measured after it was segmented using the proposed GT segmentation method (red), an alternative GT segmentation method (blue), and the MS method (black) using various parameter values such as k = 0 (solid), k = 0.2 (dashed) and k = (-0.2) (dotted). The insets show representative optical slices of the prophase nucleus with the segmentation contours of MS k = 0 (red) and GT (green). The holes of the MS resulted in different volume measurements at 4 and 5 minutes. The nucleus is in prophase (4-5 min), prometaphase (7 min), metaphase (8-9 min), anaphase (10 min) and telophase (11-13 min).

To determine whether machine segmentation artefacts cause remarkable changes in measured volumes, dividing nuclei were traced throughout mitosis. The high H2Av-GFP intensity gradient inside a prophase nucleus led to unfilled holes in segmenting a syncytial blastoderm nucleus with MS (Figure 5), resulting in different volume measurement between GT and MS segmentation.

#### 3D nucleus segmentation to detach free histone

The individual and manual specification of several thousand ROI contours of a segmented image stack is a flexible, but slow and laborious method, prone to human error. 3D object-based segmentation speeds up the process and the user can semi-automatically segment a whole object by using a single threshold. A typical nucleus spreads over ~10 to ~30 slices, thus by choosing a 3D object-level *θ_min _*many ROIs can be segmented simultaneously. Selecting an optimal *θ_min _*is the main user task after the x, y, z coordinates of a seed pixel are chosen. The middle row image series of Figure (3e, f, g, h) guides the viewer through the process via the 3D segmentation of a metaphase nucleus. The volume-rendered 3D view (Figure 3e) shows a heterochromatin region surrounded by free histone from below that has been spuriously attached because of an incorrectly low *θ_min _*value (Figure 3f). The optimal *θ_min _*removes the free histone and at the same time preserves the shape of the mitotic nucleus (Figure 3g), resulting in the correctly segmented optical slices (Figure 3h).

**Figure 6:**
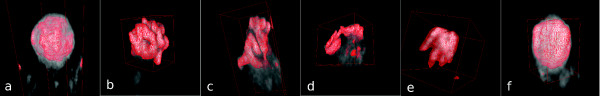
**Nuclei in various phases**. Nuclei of a *Drosophila *embryo in postcellular blastoderm developmental stage. (a) Interphase volume (V) = 214.2 *μm*^3^, (b) prophase V = 77.1 *μm*^3^, (c) metaphase V = 62.2 *μm *^3^, (d) anaphase A V = 58.8 *μm*^3^, (e) anaphase B V = 27.7 *μm*^3^, (f) telophase V = 57.1 *μm*^3^.

As a result, nuclei are segmented in interphase and various mitotic phases such, as prophase, metaphase, anaphase A, anaphase B and telophase, respectively (See Figure 6 and additional files 5a-f). This module also enables the user to distinguish between axial and lateral 3D object fusions (unpublished data).

#### Segmentation of a low contrast embryonic image stack

The brightly stained central yolk mass of the *Drosophila *embryo is still overlapping with the periplasm during nuclear cycle 10 in the syncytial blastoderm, which lasts until the the depth of yolk-free periplasm increases dramatically at the expense of the central yolk region in nuclear cycle 13 [39]. As a result, the contrast of a whole image stack becomes low owing to the uniform intensity of foreground and background pixels (Figure 3i). Global thresholding (Figure 3j) gives visually unacceptable segmentation results. The individual 3D object-based GT specification is a segmentation method that was able to remove the noise around the nuclei (Figure 3k) and create a labelled image stack (Figure 3l).

### Case study 2: 3D segmentation of Drosphila brain

**Figure 7:**
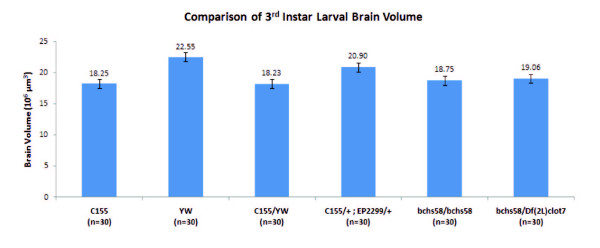
**Comparison of mutant Drosophila brain volumes**. Both genotypes of *bchs58/Df(2L)clot7 *and homozygous *bchs58 *have a reduction in the brain volumes of third instar larvae compared to the wild type stock yw. The *Gal 4 *driver line C155 and C155 crossed to yw were used as additional controls for C155 crossed to the *Bchs*-overexpressing EP2299 line.

In order to provide a quantitative measurement of the strength of phenotypes resulting from *bchs *gain-of-function or loss-of-function mutations, Lim and Kraut first made use of the percentages of larval RP2 motor neuron loss through the immunostaining of GFP-expressing RP2 motor neurons [13]. However, we sought to make the phenotypic measurement faster and more quantitative. Therefore in this study, we have measured the brain volumes of different late third instar *bchs *mutant larvae (Figure 7) by labelling the dissected whole larval brain with rhodamine-conjugated phalloidin, which recognizes F-actin. The *bchs58 *allele is a nonsense mutation that encodes for a truncated protein, while Df(2L)clot7 is a deficiency on the left arm of chromosome 2. As shown in Table 1, both genotypes of *bchs58*/*Df*(*2L*)*clot7 *and homozygous *bchs58 *have a 15% to 17% reduction in the brain volumes of the third instar larvae, when compared with the brains of the larvae from the genetic background of the *bchs58 *allele mutants (YW).

**Table 1:** Comparison of mutant Drosophila brain volumes

Genotype 1	Genotype 2	p-value	volume difference^a^[%]
C155/yw	C155/+; EP2299/+	< 0.001	+ 14.6%
YW	*bchs58/bchs58*	< 0.001	- 16.9%
YW	*bchs58/Df(2L)clot7*	< 0.001	- 15.5%
*bchs58/bchs58*	*bchs58/Df(2L)clot7*	0.641	NA
C155/+; EP2299/+	*bchs58/bchs58*	< 0.001	- 10.3%
C155/+; EP2299/+	*bchs58/Df(2L)clot7*	< 0.01	- 8.8%

Using Gebiss we show that both *bchs *null mutant combinations of *bchs58*/*Df(2L)clot7 *and homozygous *bchs58 *have a reduction in the brain volumes of third instar larvae, when compared with the background genotype YW brains as a control. ^a^The difference in brain volume of Genotype 2 in comparison with Genotype 1 as the reference control. NA: Non-applicable.

This reduction in *bchs *mutant brain volume is statistically significant (unpaired Student's t test, p < 0.001) as compared with the YW control and it is in agreement with our previous phenotypic quantification method of RP2 motor neuron loss [13]. In addition, the smaller reduction in *bchs *mutant brain volume at the third instar larval stage in comparison with an estimated 40% reduction in adult *bchs *mutant brain volume [14] may be explained by the longer duration of the adult stage which allows progressive neurodegeneration to occur. From this brain volumetric analysis, there is no significant difference between the two different *bchs *null alleles, *bchs58/Df(2L)clot7 *and homozygous *bchs58*, indicating that the *bchs58 *allele is, as expected from earlier studies [13], acting as a null allele with respect to *bchs*' effect on brain volume. The reduction in brain volume of *bchs *null larvae is consistent with the earlier observation made in adult animals [14] and suggests that the overall level of degeneration in the larval brain can be assessed using the Gebiss method of volumetric analysis.

Conversely, over-expression of the *bchs *using the EP2299 line in conjunction with the C155 *Gal4 *driver in the central nervous system resulted in an increase of 14.6% brain volume when compared with the control, C155/yw. Together with the observed shrinkage in brain volume of loss of function in *bchs *mutants, these results suggest that *Bchs *may have some role in determining cellular volume and/or proliferation in the brain.

### Segmentation performance evaluation

**Figure 8:**
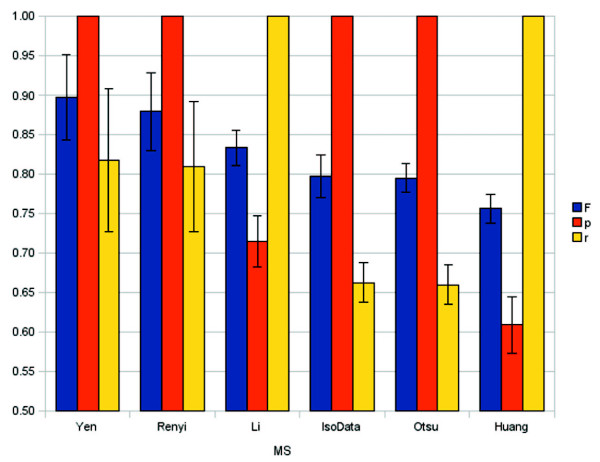
**Machine segmentation performances on Drosophila brain segmentation**. Performance evaluation of six MS algorithms segmenting *Drosophila *brain stacks (n = 5). All algorithms calculate a single global threshold. When that threshold is smaller than that of GT, there will not be any false positive voxel which leads to 100% precision (p = 1, orange) in case of Yen, Rényi, IsoData and Otsu methods. When the MS threshold is calculated to be larger than that of GT, there will not be any false negative voxel resulting in 100% recall (r = 1, yellow) in the case of the Li and Huang methods. F-measure values are represented by the blue bars. Error bars represent standard deviation.

To test the performance of 3D segmentation methods, we applied six different automated MS algorithms to segment *Drosophila *brain stacks: Yen's method [40], Rényi entropy [41], Li's minimum cross entropy [42], Huang's fuzzy thresholding [43], Ridler and Calvard's iterative Isodata method [44] and Otsu's thresholding [45] (Figure 8). The segmentation result image stacks can be found in additional file 6: "Visual segmentation performance evaluation". All algorithms are implemented under ImageJ by Gabriel Landini, and compute a global threshold from the stack image to segment the stack, with the limitation that only 8-bit image stacks are supported. The advantage is that no manual parameter entry is required. The computed threshold is lower, equal to or higher than that of ground truth.

Therefore, the segmented brain stacks resulted in greater, equal to or smaller volumes, respectively. MS volumes greater than GT lack any false negative pixels, thus leading to r = 1 recall in the case of Li and Huang segmentation. Conversely, MS volumes smaller than GT lack any false positive pixels, thus resulting in p = 1 precision at Yen, Rényi, Isodata and Otsu methods.

**Figure 9:**
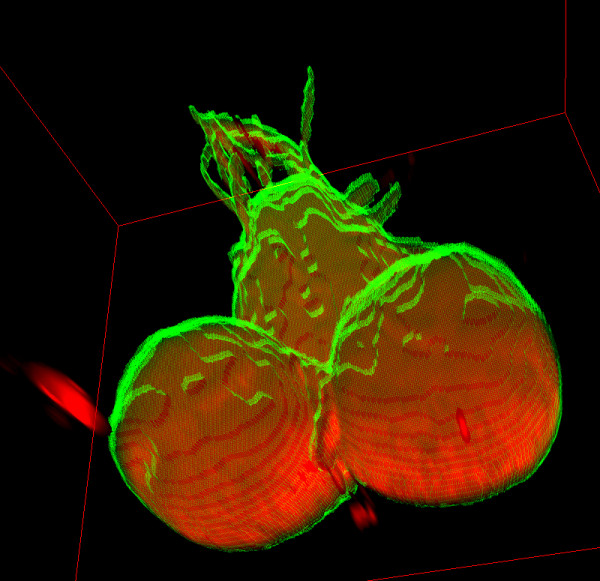
**Bchs58/c17 neurodegenerative mutant Drosophila brain GT segmentation visualised in 3D**. *Drosophila *central nervous system segmented voxels (green isosurface mesh) superimposed on original voxel intensities (red volume rendering), the volume is 22.38 × 10^6 ^*μm *^3^.

The 8-bit image stacks had histograms shifted to darker voxel intensities. We used the global threshold value 23 to create GT (Figure 9). However, global threshold values between 19 and 28 gave F-measures 0.95 and above, indicating that the brain contour was not sharp.

Huang's fuzzy thresholding belongs to the algorithms that uses Shannon's entropy function and segments based on attribute similarity. Measuring the similarity between the original and the binary image stack, it determined the threshold by minimising the measure of fuzziness. Including average foreground and background intensities, the algorithm computed a low MS threshold value in our dark stacks, resulting in numerous false positive voxels and the poorest mean F-measure, which is still above 0.75.

Although widely used popular methods in imaging, the performance of the clustering-based segmentation algorithms of Isodata and Otsu performed less well than the others. Both methods assume that a stack has two grey intensity maxima and partition the histogram of an image stack into two classes, based on intra-class variance minimisation and inter-class variance maximisation. The Otsu method searches for the optimal threshold globally, whereas the Isodata does this locally, thus these methods result in almost identical thresholds. These algorithms work optimally with a bimodal histogram, where the number of voxels are similar in both the foreground and the background class. One reason for the weaker segmentation performance is that the number of black background voxels affect the threshold computed by those two algorithms. Also the background and foreground intensity modes are not separated sharply in the histograms of our brain stacks.

Among the tested MS algorithms, the three entropy-based segmentation methods performed the best. Li's method is based on cross-entropic thresholding. It has a significantly reduced computational requirement compared with the exhaustive search method but it tends to calculate a lower threshold value than that of GT.

We found that Yen's method and Rényi's entropy performed the best among the algorithms that were tested. The two algorithms are similar and performed similarly well. Both belong to Shannon-entropy maximisation-based segmentation methods [46], originating from one-dimensional entropic thresholding introduced by Pun in 1981 [47].

## Conclusions

In this paper, we present Gebiss, a new software for quantitative 3D segmentation performance evaluation. Gebiss was designed to be a productive and user friendly tool for ground truth creation, and it includes a benchmarking module that objectively evaluates a 3D segmentation. The package was developed as a plugin for ImageJ, is platform independent and can be freely downloaded from http://imaging.bii.a-star.edu.sg/projects/gebiss/. Gebiss was successfully used in various biological tasks.

## Availability and requirements

**Project name: **Gebiss

**Project home page: **http://imaging.bii.a-star.edu.sg/projects/gebiss/

**Operating system: **platform independent

**Programming language: **Java

**Other requirements: **ImageJ 1.43 m or higher, Java 1.6.0_16 or higher

**License: **GNU GPLv3

## Authors' contributions

JKV carried out the design and implementation of the software, analysed the images and wrote the manuscript. NWT participated in the implementation of the software, CLP coordinated the implementation of the software, KCY participated in the implementation of the software, JSPL carried out the *Drosphila *brain experiment, RK conceived and coordinated the *Drosphila *brain experiment, MW conceived and coordinated the study. All authors read and approved the final manuscript.

## Supplementary Material

Additional file 1 **ImageJ macro for GT contour visualization**. ImageJ macro file in Unix text format. Before running, it requires two input image stacks to be opened. An original image stack, converted into RGB format and renamed as "orig" as well as its matching binary stack renamed as "bin" with black foreground and white background pixels. For the macro operation see the text. Click here for file

Additional file 2 **3D GT contour visualization**. Compressed, spatially calibrated RGB image stack that can be opened by standard ImageJ. GT segmentation contours (green) are superimposed on the original, low contrast image slices. By simultaneously visualising both the foreground and background pixels, the user can easily check the ground truth segmentation. The stack's xyz dimensions are 133.3 *μm *× 133.3 *μm *× 29.9 *μm*. Click here for file

Additional file 3 **Numerical output of the Gebiss benchmarking module**. Quantitative machine segmentation benchmark results demonstrated on a low contrast image stack of a *Drosophila *embryo in syncytial blastoderm developmental stage, containing 68 slices. Data in columns represents the evaluation of each optical slices. The slice numbers are indicated in the table header, followed by measures of each individual slices respectively: number of GT and MS objects (if any), number and labels of merged and split objects (if any), number and labels of false positive and false negative MS objects (if any). Click here for file

Additional file 4 **Visual output of the Gebiss benchmarking module**. Compressed, spatially calibrated image stack. It can be opened using ImageJ. It horizontally combines the MS (left) and the GT (right) 32-bit labelled stacks in an easily comparable manner. Demonstrated on a low contrast image stack containing 68 slices. Click here for file

Additional file 5 **Nuclei in various phases**. Six animated GIF movies in one compressed file. After uncompressing, those can be opened by ImageJ or an internet browser. The movies show nuclei of a *Drosophila *embryo in postcellular blastoderm developmental stage. (a) Interphase volume (V) = 214.2 *μm*^3^, (b) prophase V = 77.1 *μm*^3^, (c) metaphase V = 62.2 *μm*^3^, (d) anaphase A V = 58.8 *μm*^3^, (e) anaphase B V = 27.7 *μm*^3^, (f) telophase V = 57.1 *μm*^3^. Click here for file

Additional file 6 **Visual segmentation performance evaluation**. ZIP compressed, spatially calibrated, segmented binary image stacks, that can be opened by standard ImageJ. The images represent the 3D segmentation results discussed in the section "Segmentation performance evaluation". File names indicate the machine segmentation that resulted the given stack. Click here for file
