# Supplementary figures and images for: Gebiss: an ImageJ plugin for the specification of ground truth and the performance evaluation of 3D segmentation algorithms
Source: BMC Bioinformatics. 2011 Jun 13;12:232. doi: 10.1186/1471-2105-12-232 (PMC3225128; doi:10.1186/1471-2105-12-232)

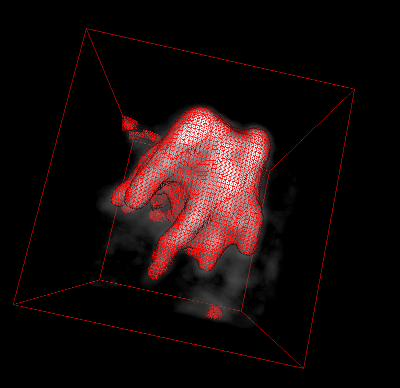

Supplement: Additional file 5 — Nuclei in various phases. Six animated GIF movies in one compressed file. After uncompressing, those can be opened by ImageJ or an internet browser. The movies show nuclei of a Drosophila embryo in postcellular blastoderm developmental stage. (a) Interphase volume (V) = 214.2 μm3, (b) prophase V = 77.1 μm3, (c) metaphase V = 62.2 μm3, (d) anaphase A V = 58.8 μm3, (e) anaphase B V = 27.7 μm3, (f) telophase V = 57.1 μm3. [file 1471-2105-12-232-S5.ZIP › e_anaphaseB_27.7um3.gif]

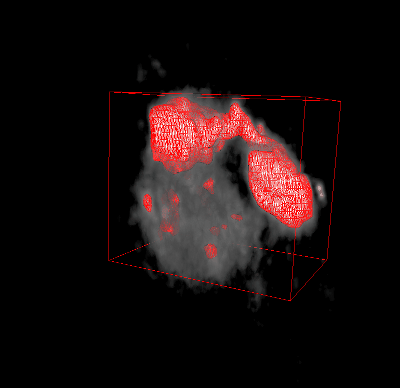

Supplement: Additional file 5 — Nuclei in various phases. Six animated GIF movies in one compressed file. After uncompressing, those can be opened by ImageJ or an internet browser. The movies show nuclei of a Drosophila embryo in postcellular blastoderm developmental stage. (a) Interphase volume (V) = 214.2 μm3, (b) prophase V = 77.1 μm3, (c) metaphase V = 62.2 μm3, (d) anaphase A V = 58.8 μm3, (e) anaphase B V = 27.7 μm3, (f) telophase V = 57.1 μm3. [file 1471-2105-12-232-S5.ZIP › d_anaphaseA_58.8um3.gif]

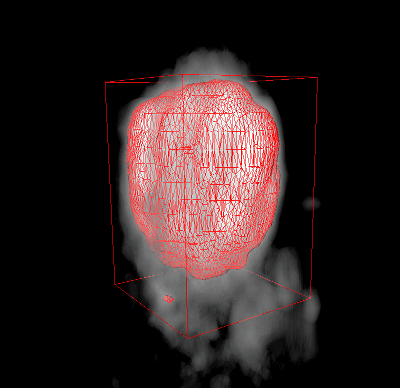

Supplement: Additional file 5 — Nuclei in various phases. Six animated GIF movies in one compressed file. After uncompressing, those can be opened by ImageJ or an internet browser. The movies show nuclei of a Drosophila embryo in postcellular blastoderm developmental stage. (a) Interphase volume (V) = 214.2 μm3, (b) prophase V = 77.1 μm3, (c) metaphase V = 62.2 μm3, (d) anaphase A V = 58.8 μm3, (e) anaphase B V = 27.7 μm3, (f) telophase V = 57.1 μm3. [file 1471-2105-12-232-S5.ZIP › f_telophaseB_57.1um3.gif]

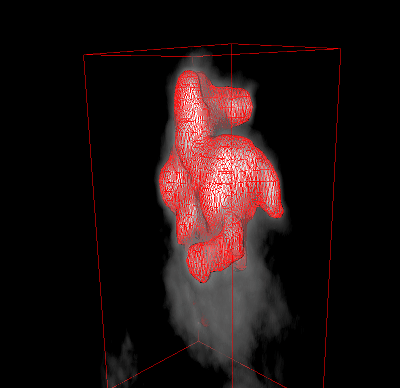

Supplement: Additional file 5 — Nuclei in various phases. Six animated GIF movies in one compressed file. After uncompressing, those can be opened by ImageJ or an internet browser. The movies show nuclei of a Drosophila embryo in postcellular blastoderm developmental stage. (a) Interphase volume (V) = 214.2 μm3, (b) prophase V = 77.1 μm3, (c) metaphase V = 62.2 μm3, (d) anaphase A V = 58.8 μm3, (e) anaphase B V = 27.7 μm3, (f) telophase V = 57.1 μm3. [file 1471-2105-12-232-S5.ZIP › c_metaphase_62.2um3.gif]

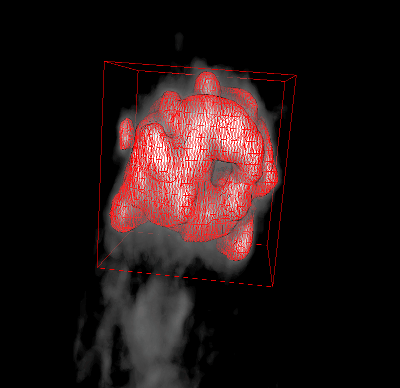

Supplement: Additional file 5 — Nuclei in various phases. Six animated GIF movies in one compressed file. After uncompressing, those can be opened by ImageJ or an internet browser. The movies show nuclei of a Drosophila embryo in postcellular blastoderm developmental stage. (a) Interphase volume (V) = 214.2 μm3, (b) prophase V = 77.1 μm3, (c) metaphase V = 62.2 μm3, (d) anaphase A V = 58.8 μm3, (e) anaphase B V = 27.7 μm3, (f) telophase V = 57.1 μm3. [file 1471-2105-12-232-S5.ZIP › b_prophase_77.1um3.gif]

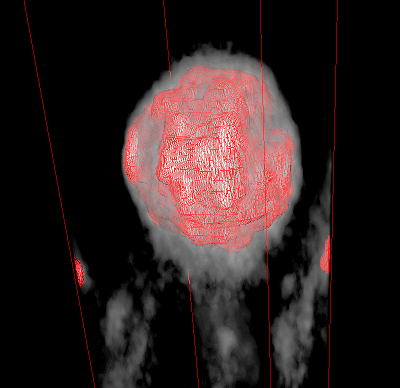

Supplement: Additional file 5 — Nuclei in various phases. Six animated GIF movies in one compressed file. After uncompressing, those can be opened by ImageJ or an internet browser. The movies show nuclei of a Drosophila embryo in postcellular blastoderm developmental stage. (a) Interphase volume (V) = 214.2 μm3, (b) prophase V = 77.1 μm3, (c) metaphase V = 62.2 μm3, (d) anaphase A V = 58.8 μm3, (e) anaphase B V = 27.7 μm3, (f) telophase V = 57.1 μm3. [file 1471-2105-12-232-S5.ZIP › a_interphase_214.2um3.gif]
